# Current and future advances in practice: a practical approach to the diagnosis and management of primary central nervous system vasculitis

**DOI:** 10.1093/rap/rkad080

**Published:** 2023-11-20

**Authors:** Mats Junek, Kanjana S Perera, Matthew Kiczek, Rula A Hajj-Ali

**Affiliations:** Department of Medicine, Division of Rheumatology, McMaster University, Hamilton, ON, Canada; Department of Medicine, Division of Neurology, McMaster University, Hamilton Health Sciences, Hamilton, ON, Canada; Department of Rheumatic and Immunologic Disease, Center for Vasculitis Care and Research, Cleveland Clinic, Cleveland, OH, USA; Department of Neuroradiology, Imaging Institute, Cleveland Clinic, Cleveland, OH, USA

**Keywords:** vasculitis, central nervous system, brain biopsy, neuroradiology, collaborative care

## Abstract

Primary CNS vasculitis (CNSV) is a rare, idiopathic autoimmune disease that, if untreated, can cause significant morbidity and mortality. It is a challenging diagnosis due to multiple mimics that can be difficult to differentiate, given that the CNS is an immunologically privileged and structurally isolated space. As such, diagnosis requires comprehensive multimodal investigations. Usually, a brain biopsy is required to confirm the diagnosis. Treatment of CNSV involves aggressive immunosuppression, but relapses and morbidity remain common. This expert review provides the reader with a deeper understanding of presentations of CNSV and the multiple parallel diagnostic pathways that are required to diagnose CNSV (and recognize its mimics), highlights the important knowledge gaps that exist in the disease and also highlights how we might be able to care for these patients better in the future.

Key messages

- Primary CNS vasculitis (CNSV) is a rare but potentially devastating diagnosis that requires clinical suspicion and comprehensive investigation to diagnose.
- A clinician needs to assess for atherosclerotic, embolic disease, infectious, immune, neoplastic, genetic and other disease mimics before confirming a diagnosis of CNSV.
- Advances in imaging have assisted in the diagnosis of primary CNSV; however, a brain biopsy is still the standard investigation to confirm the diagnosis.
- Treatment of CNSV requires glucocorticoids and long-term systemic immunosuppression because relapses occur.
- There are multiple knowledge gaps, but growing research initiatives will allow improvements in the diagnosis, management and outcomes for patients with CNSV.

## Introduction

CNS vasculitis (CNSV) is the presence of inflammation within the blood vessels of the brain, meninges and spinal cord. CNSV can be primary when confined to these structures (CNSV) or secondary to a systemic inflammatory process, infection or other systemic process (secondary CNSV). CNSV was first identified in the 1950s; however, there continue to be many gaps in our understanding of its pathogenesis, diagnosis and management attributable, in part, to the rarity of the illness and under-recognition of the diagnosis [1]. Accumulating cases and growing research interest, however, have established several principles of practice for the disease. This review summarizes current expert approaches to primary CNSV (referred to as CNSV throughout this manuscript), key pearls and pitfalls, and highlights how we might improve the care provided for patients with this diagnosis.

## Taxonomy and definitions in CNSV

### Criteria used in the diagnosis of CNSV

The commonly used criteria for the diagnosis of CNSV require that the patient had a history or clinical findings of an acquired neurologic deficit, which remained unexplained after a thorough initial basic evaluation and that there are classic angiographic or histopathological features of angiitis within the CNS and that there is no evidence of systemic vasculitis or any other condition to which the angiographic or pathological features could be secondary [1].

Although these criteria have never been endorsed formally for either diagnosis or classification, they continue to be used in research and clinical practice and represent a robust diagnostic philosophy. It has been increasingly recognized, however, that imaging techniques in CNSV do have limitations in differentiating CNS vasculopathies and that CNSV can only be considered definite when appropriate findings are seen on brain biopsy. It has thus been proposed that changes seen only on imaging should be considered probable and not definite CNSV [2]. Research is ongoing to understand better how advances in imaging should be incorporated into the diagnosis and management of CNSV [3, 4].

### Disease subtypes

Initial efforts to subtype CNSV focused on the mode of diagnosis and classified CNSV as either histological or radiographic subtypes. Over time, these have evolved to focus on the size of vessel affected: small vessel CNSV (svCNSV) and proximal, medium-to-large vessel CNSV (lvCNSV), which includes the intracranial carotid, intracranial vertebral, basilar and proximal branches of the cerebral arteries [5, 6]. This change in taxonomy reflects that svCNSV often has non-vascular radiographic changes, and radiographically conspicuous lvCNSV will, less frequently, have changes on brain biopsy [6–10]. Both imaging and histology are important for the diagnosis of CNSV and distinguishing these subtypes. Given that data are limited, it is currently unclear whether these subtypes represent variant presentations of CNSV or possibly different disease processes, although there is a suggestion that patients with lvCNSV are at higher risk of relapse [11–13].

## Clinical presentations

CNSV typically presents in individuals aged 40–60 years and appears to affect both sexes equally [14–16]. There are often weeks to months of prodromal symptoms, including headache, cognitive impairment, personality change and/or constitutional symptoms, followed by the onset of acute neurological changes, including strokes, encephalopathy, seizures and/or other deficits (Table 1). The most common presentation is insidious headaches and strokes, with a negative diagnostic work-up for more common secondary causes. Given that the prodromal phase also represents a presentation of myriad other conditions more common than CNSV, the diagnosis is typically not considered until there have been sufficient events to trigger an investigation for atypical disease processes or imaging suggests that a CNS vasculopathy is present. Although a post-morbid investigatory strategy is not ideal, there are currently no data that allow clinicians to risk stratify patients presenting with neurological symptoms to their probability of CNSV.

**Table 1.** Frequency of presenting features in primary CNS vasculitis, stratified as either pathologically or radiographically diagnosed central nervous system vasculitis

Manifestation/finding	All CNSV	Pathologically diagnosed CNSV	Radiographically diagnosed CNSV	Reference
**Clinical**				
Headache	51.0–52.6	53.0	57.0–59.0	[16, 66]
Focal neurological deficit	21.0–41.4	24.0–26.0	8.0–38.0	[16, 66]
Speech disorder	20.0–21.0	15.0–21.0	15.0–17.0	[16, 66]
Seizure	22.0–24.0	28.0–36	16.0	[16, 66]
Disordered cognition	41.0	54.0–55	36.0–39.0	[16, 66]
Ataxia	15.0–19.0	13.0–17.0	14.0–20.0	[16, 66]
Psychiatric disturbance	7.4–15.0	7.1–18.0	3.4–9.0	[16, 66]
Fever	5.0–12.0	5.5–16.0	5.7–9.0	[16, 66]
**Serological**				
Any CSF abnormality	69.0–75.0	75.0–91.0	55.0–65.0	[5, 16, 66]
Increased CSF white blood cells	48.0–48.2	60.0–61.0	35.0–36.7	[16, 66]
Increased CSF protein	64.8–66.4	72.0–73.6	59.0–63.6	[16, 66]
**Radiological**				
Any MRI change	97.0–97.6	97.0–98.0	97.0–98.0	[16, 66]
Infarct on MRI	53.4–60.0	34.0	63.0–68.0	[16, 66]
Leptomeningeal MRI changes	13.0–28.0	33.0–54.0	12.0–17.0	[5, 16]
Changes on angiography	54.8–78.9	0–41.0	65.7–100	[5, 7, 16]
**Histological**				
Brain biopsy positive	65.3	100	26.0	[5]
Granulomatous vasculitis	37.5	46.1	0	[5]
Lymphocytic vasculitis	84.4	80.7	100	[5]
Necrotic vasculitis	34.4	42.6	0	[5]

CNSV: central nervous system vasculitis; CSF: cerebrospinal fluid.

Less frequent clinical presentations of CNSV include a more indolent and slowly progressive presentation of cognitive and/or neurological changes; presentations of inflammatory mass-like lesions with symptoms related to mass effect; and spinal cord lesions presenting with spinal syndromes [17, 18]. Case reports of CNSV presenting with new-onset refractory status epilepticus and various cranial neuropathies have also been published [18, 19].

## An approach to the diagnosis of CNSV

Initial diagnoses of CNSV were limited to autopsies and brain biopsies that were performed in patients with episodic, progressive neurological defects [20, 21]. Modern diagnoses of CNSV can be made much earlier in the disease course with the introduction of new imaging and biopsy techniques, however, it remains a challenging diagnosis to confirm. Four diagnostic pathways should be pursued in parallel when considering a possible diagnosis of CNSV:

(i) demonstrate that there are radiological changes consistent with a CNS vasculopathy with vascular and/or parenchymal features suggestive of a vasculitic process;

(ii) demonstrate that there is a neuroinflammatory process;

(iii) rule out mimics of CNSV; and

(iv) consider a brain biopsy.

### Demonstrate that there is a CNS vasculopathy with features suggestive of an inflammatory aetiology

Owing to the non-specific symptoms of CNSV, the first suggestion that this is the underlying process is often when neuroimaging demonstrates that there is a CNS vasculopathy with features suggesting an inflammatory aetiology based on the distribution of vessels affected, changes in the vessel wall (VW) and lumen, and parenchymal abnormalities. Assessment of the vessel lumen is performed via CT angiography (CTA), magnetic resonance angiography (MRA) and/or more invasive digital subtraction angiography (DSA). These modalities are useful first-line evaluations but provide information only about the vessel lumen [22].

Digital subtraction angiography has the highest resolution of all modalities and is the most sensitive for small to medium vessels [23]. CTA offers the highest non-invasive resolution of larger vessels for luminal narrowing or occlusion, but MRI with MRA is the most sensitive non-invasive modality overall, because it captures both vascular and parenchymal changes [24]. In svCNSV, MRI plus MRA may not sufficiently assess small, distal vessels that are affected by the disease process. In these cases, DSA should be considered to evaluate these vessels, although a negative DSA will not rule out CNSV, because these vessels may still be too small to resolve, and a biopsy may be needed to determine the final diagnosis [24]. Secondary parenchymal changes seen on MRI supportive of a CNS vasculopathy can include infarcts in multiple vascular territories of varying ages, meningeal enhancement, hyperintense foci on T2 and fluid-attenuated inversion recovery (FLAIR) sequences, microhaemorrhage attributable to small vessel vasculitis and, rarely, tumour-like lesions [17, 24]. Meningeal and parenchymal changes are seen more frequently in individuals with svCNSV; ischaemic lesions in lvCNSV [5, 25]. It is important to note, however, that these findings are non-specific and can be considered only in conjunction with clinical evaluation and vascular imaging.

**Figure 1.**
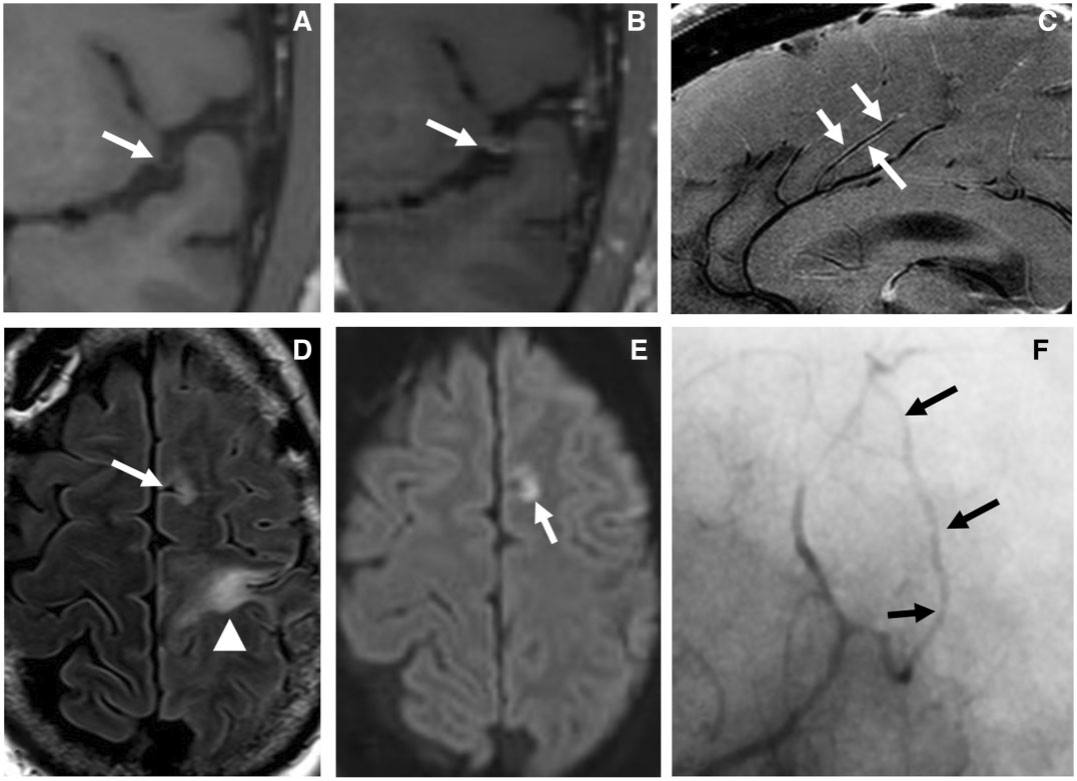
Radiographic findings of primary CNS vasculitis. (**A**, **B**) Pre- (A) and post-gadolinium (B) T1 SPACE high-resolution MR-VWI shows circumferential vessel wall enhancement (B, arrow) in a patient with suspected CNSV. (**C**) 7 T MR-VWI following gadolinium administration shows diffuse pericallosal artery branch wall enhancement in biopsy-proven CNSV (arrows). (**D**) FLAIR MRI showing evolving signal changes from parenchymal insults of varying ages, including subacute (arrowhead) and acute (arrow). (**E**) diffusion-weighted MRI in the same patient as (E) shows that the paramedian frontal insult is acute. (**F**) Digital subtraction angiography in the same patient showing subtle areas of distal anterior cerebral artery territory beaded luminal irregularity. CNSV: CNS vasculitis; FLAIR: fluid-attenuated inversion recovery; VW: vessel wall

**Figure 2.**
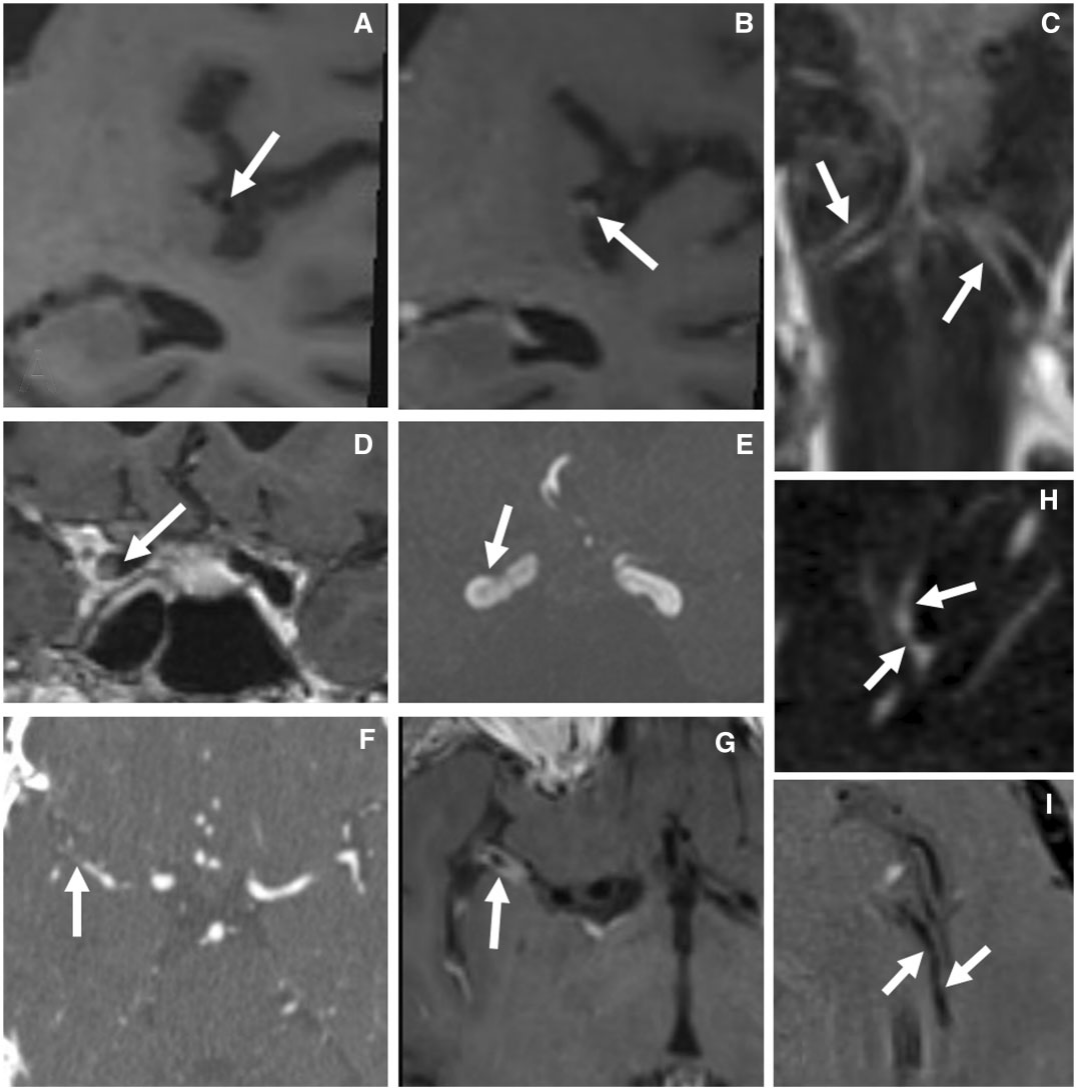
Mimics of CNS vasculitis seen with magnetic resonance vessel wall imaging (MR-VWI). (**A**, **B**) Before (A) and after (B) T1 SPACE high-resolution MR-VWI showing an area of eccentric M2 wall thickening and enhancement (arrow) related to non-inflammatory atherosclerotic changes. (**C**) Post-gadolinium coronal T1 SPACE MR-VWI showing vasa vasorum enhancement of the proximal V4 segments in atherosclerotic disease (often mistaken as inflammation). (**D–G**) Images representing distal M1 thrombosis with a finding of circumferential enhancement (**G**) secondary to thrombectemy: area of apparent filling defect in the right cavernous ICA segment (**D**, arrow) owing to flow-related artefact, with E showing patency on time-of-flight (TOF) magnetic resonance angiography; CTA demonstrating acute distal right M1 occlusion (F, arrow) and G showing MR-VWI following mechanical thrombectomy, with circumferential enhancement thought to be related to mechanical manipulation and disruption of the endothelium. (**H**, **I**) Images from a patient with reversible cerebral vasoconstriction syndrome with multifocal areas of luminal narrowing (H) of the proximal M2 on CTA (arrows) and absence of vessel wall enhancement (I) in the same region on MR-VWI suggesting a diagnosis of reversible cerebral vasoconstriction syndrome. CNSV: CNS vasculitis; CTA: CT angiography

MR vessel wall imaging (MR-VWI) uses high-resolution MRI machines, contrast, and advanced signal processing that optimizes contrast-to-noise ratios to allow for resolution of the vessel wall, which has ∼1/10^th^ the diameter of the lumen [26, 27]. MR-VWI has quickly become integral in the evaluation of intracerebral vasculopathy, but is not available in many medical centres. The characteristics of the vessel wall changes and the pattern of vessels affected may also offer clues concerning the aetiology of the changes [23]. The most common pattern of vascular change seen using MR-VWI in inflammatory CNS vasculopathies is scattered foci of smooth, homogeneous circumferential involvement (Fig. 1) [26, 28]. Where there is suspicion for lvCNSV (or other large vessel vasculopathies), one must also be aware of increased enhancement from the vasa vasorum enhancement at sites where vessels penetrate the dura (V3–V4 segment of vertebral artery and cavernous/supraclinoid internal carotid artery), traversing veins mimicking wall enhancement, and slow-flow-related artefacts, which are commonly reported erroneously as inflammatory changes [26, 27]. MR-VWI enhancement in intracranial atherosclerotic disease, a common mimic, is usually eccentric and irregular (Fig. 2). There can be overlap in the appearance between the two, however, as intracranial atherosclerotic disease can be circumferential [28], and the two conditions can also co-exist.

MR-VWI is a promising modality that helps to reveal the changes of CNSV, but it complements and does not replace other imaging techniques, clinical testing or serological/cerebrospinal fluid (CSF) analysis. As a burgeoning imaging modality, it is not universally available, and non-MR-VWI, CTA, MRA and DSA can still provide valuable diagnostic information. Early in the disease course, changes on MR-VWI may be subtle, and parenchymal abnormalities, seen in virtually all cases of CNSV, may be the only radiographic indicator of disease. Given that parenchymal MRI changes are more sensitive for CNSV, normal MRI parenchymal imaging (instead of normal VW-MRI), alongside normal CSF analysis, has high negative predictive value for a diagnosis of CNSV [29].

### Demonstrate that there is a neuroinflammatory process

Demonstrating a neuroinflammatory state has historically been considered a hallmark of the diagnosis of CNSV. CRP and ESR are typically normal and, if elevated, should prompt evaluation for systemic inflammatory, infectious or thrombotic processes [16]. Evaluation of CSF is more sensitive and specific for a neuroinflammatory process; changes are seen in 75–81% of all patients with CNSV, and a normal CSF should be considered highly suggestive of an alternative diagnosis [16, 18, 30]. Limited data suggest that abnormalities might be seen more frequently in svCNSV (83%) than in those with lvCNSV (55%); many of these patients did not have a biopsy to confirm the diagnosis, and mimics might have been included in the lvCNSV cohort [16]. The most common abnormalities seen are a mild increase in CSF protein and/or pleocytosis. Although less sensitive than elevated protein, pleocytosis might be more specific in discriminating CNSV from other diseases, although patients with pleocytosis should first be assessed for a possible CNS infectious process [31]. Non-matching CSF oligoclonal bands and/or a high IgG index is found in ∼25% of CNSV and, in the absence of other diagnoses, can strengthen the diagnosis [32, 33].

### Rule out mimics of CNSV

Systemic diseases and mimics typically fall into five differential categories: immune-mediated inflammatory diseases, malignancy, non-inflammatory vasculopathies, infectious vasculitis and other (Table 2). The most common mimics of CNSV are premature atherosclerosis and non-inflammatorycerebral vasculopathies. Specific mimics that frequently arise with the possibility of CNSV and how to differentiate them are presented in Table 3.

**Table 2.** Differential diagnoses of primary central nervous system vasculitis

Immune-mediated inflammatory diseases	Malignancy
ANCA-associated vasculitis	Lymphoma
ANA-mediated autoimmune diseases (SLE, SS, etc.)	LeukaemiaAngioendotheliomatosis
PAN	Atrial myxoma
Behçet’s syndrome	Lymphomatoid granulomatosis
GCA and Takayasu arteritis	**Infection**
Sarcoidosis	Viral: HIV, HSV, VZV, EBV, CMV, West nile virus, COVID-19
Cryoglobulinemic vasculitis	Bacterial: Lyme, syphilis, *Mycobacteria*, *T. whippelii*, *Bartonella* spp., *M. pneumoniae*, *Rickettsia*
Rheumatoid vasculitis	Fungal: *Aspergillus*, *Candida* spp., *Coccidioides* spp., *Cryptococcus* spp.
Combined variable immunodeficiency (CVID)-associated CNS granulomatous disease	Parasitic: *P. falciparum*, *T. gondii*
Susac syndrome	
Autoimmune encephalopathies	
Demyelinating disease	

**Vasculopathies**	**Other**

Fibromuscular dysplasia	Cerebral autosomal dominant/recessive arteriopathy with sub-cortical infarcts and leucoencephalopathy (CADASIL/CARASIL)
Moyamoya disease	Thrombotic thrombocytopenic purpura
Segmental mediolar arteriopathy	Retinal vasculopathy with cerebral leukoencephalopathy (RVCL)
Reversible cerebrovascular syndrome	Adenosine deaminase 2 (ADA2) deficiency
Intracerebral atherosclerotic disease	Haemophagocytic lymphohistiocytosis
Radiation vasculopathy	Paraneoplastic vasculopathy/encephalopathy

**Table 3.** Key mimics of primary central nervous system vasculitis

Clinical features	Serological and CSF findings	Radiographic and histological appearance
**Reversible cerebral vasoconstriction syndrome** [67]		

Demographics: females aged 30–50 years Presentation: thunderclap headache, often triggered by stimulants/stressors/other factors; may be associated with neurological deficits and/or seizure.	Serology: unremarkable CSF: unremarkable, may see transient abnormalities secondary to haemorrhage or infarction. Pleocytosis is rarely seen in acute stroke	Angiography demonstrates ‘sausage on a string’ on multiple vessel beds bilaterally that resolves on serial imaging. Parenchyma may show secondary haemorrhage or ischaemic lesions. MR-VWI does not show thickening or enhancement.

**Atheroembolic disease**		

Demographics: affects both sexes, incidence increases with age, starting at 50 years Presentation: stepwise neurological defects, typically no prodrome. Patients have cardiovascular risk factors.	Serology: may have increased blood lipids, lipoprotein(a) CSF: unremarkable, may see transient abnormalities secondary to haemorrhage or infarction, or elevated protein if concurrent diabetes. Pleocytosis is rarely seen in acute stroke	CT/MRI demonstrates ischaemic lesions with classic evolution, periventricular white matter changes. CTA will show plaque. MR-VWI may demonstrate eccentric changes from intracranial atheromatous disease.

**Infectious vasculitis (commonly varicella zoster, herpes simplex, tuberculosis)** [34]		

Demographics: more common in the elderly or immunosuppressed. Presentation: variable, including subacute headache, encephalopathy and neurological defects (often cranial nerve palsy).	Serology: increased inflammatory markers, leucocytosis, serology and/or cultures suggestive of infection. CSF: increased protein, pleocytosis, positive serology for infectious agent.	Can be identical to CNSV on MRI/MRA/CTA; may demonstrate parenchymal lesions and white matter changes incongruent with age. Tissue culture of biopsy material may demonstrate the causative organism.

**Intracranial lymphoma** [68]		

Demographics: aged 50–70 years. Presentation: focal neurological deficits, history of AIDS and/or solid organ transplantation.	Serology: unremarkable. CSF: flow cytometry/cytology may demonstrate abnormal lymphocytic populations.	Typically, central tumour-like lesions with avid enhancement and low apparent diffusion coefficient with a predilection for the periventricular region. Meningeal enhancement, vessel wall enhancement and non-specific white matter changes. Histology demonstrates malignancy.

**CNSV secondary to immune-mediated inflammatory diseases**		

Demographics: based on underlying immune-mediated inflammatory disease. Presentation: skin changes such as livedo reticularis or photosensitivity, oral ulcers, RP, sicca symptoms.	Serology: presence of autoantibodies, leucopenia/thrombocytopenia. CSF: CSF–blood paired oligoclonal bands; increased protein and/or pleocytosis, increased CSF IgG index.	Can be identical to CNSV; may demonstrate parenchymal lesions and white matter changes incongruent with age.

**CNS granulomatous disease associated with common variable immunodeficiency (CVID)** [69]		

Demographics: variable age, more common in females. Presentation: subacute onset of headaches, neurological deficits, seizures. Known history of CVID and infections.	Serology: hypoglobulinaemia, leucopenia. CSF: typically unremarkable; may have slight increase in CSF protein and/or pleocytosis.	Mass lesions, leptomeningeal enhancements. Histology demonstrates angiocentric granulomatous changes.

**Neurosarcoidosis** [70]		

Demographics: males more commonly affected, age 40–60 years. Presentation: variable presentation of headaches, neurological deficits, seizures, neurocognitive changes. Cranial neuropathies, sinus and ocular diseases are common.	Serology: non-specific; may see elevated angiotensin-converting enzyme, vitamin D, calcium, cytopenias. CSF: may see low glucose, mild increase in CSF protein, pleocytosis and/or increased CSF angiotensin-converting enzyme and soluble IL2R.	Parenchymal changes, mass lesions and vessel wall enhancement may be seen. Nodular enhancement of meninges and cranial nerves may be seen. Pulmonary changes and lymphadenopathy are common. Histology demonstrates non-caseating granulomas.

**Autoimmune/paraneoplastic encephalopathy** [71]		

Demographics: variable, depending on malignancy. Presentation: subacute/acute seizures, encephalopathy, autonomic derangement, may have features of malignancy.	Serology: Autoantibodies may be seen, but have variable correlation with CNS disease. CSF: autoantibodies are strongly suggestive of disease.	Parenchymal changes, often in stereotyped distribution commonly involving the mesial temporal lobes; minimal evidence of vasculitis. Imaging may demonstrate a primary malignancy elsewhere in the body.

**Susac syndrome [** 41 **]**		

Demographics: females are more commonly affected, typically aged 20–40 years. Presentation: subacute triad of new changes in vision, hearing loss and encephalopathy. Patients may present with the triad sequentially or may have an incomplete triad.	Serology: unremarkable, may have low-grade elevation of inflammatory markers. CSF: may demonstrate low-grade increased protein and/or leucocytosis.	Parenchymal brain lesions, stereotyped ‘snowball’ and ‘icicle’ lesions in corpus callosum using MRI. Interfoliar leptomeningeal enhancement on MR-VWI. Cochlear enhancement has also been described.

CSF: cerebrospinal fluid; CTA: CT angiography; MRA: magnetic resonance angiography; MR-VWI: magnetic resonance vessel wall imaging; VW: vessel wall.

Clinical evaluation for mimics of CNSV includes a comprehensive history and examination. Patient demographics in addition to the pace and progression of neurological deficits can often provide helpful indications of CNSV mimics (e.g. the presence of a thunderclap headache suggesting reversible cerebral vasoconstriction syndrome compared with insidious headaches typical of CNSV). Intracranial manifestations of systemic inflammatory diseases rarely occur in isolation, and findings suggestive of an inflammatory process elsewhere in the patient should direct the clinician to consider these diagnoses. These can include small, medium and large vessel vasculitis, non-vasculitic systemic inflammatory diseases (e.g. SLE) and sarcoidosis. Equally important are travel, exposure and infectious histories; even remote exposures to tuberculosis or HIV can demonstrate reactivation with vasculitis [34]. Coronavirus disease 2019 (COVID-19) infection should be ruled out, giving its neurological complications, which can mimic CNSV. Drug exposure can also readily lead to vasculitis and can easily be overlooked. Finally, a family history of atherosclerotic disease or similar symptoms can indicate small vessel genetic diseases, including cerebral autosomal dominant or recessive angiopathy with subcortical infarcts and leucoencephalopathy (CADASIL or CARASIL) and others [35, 36].

Biochemical testing should include serology for autoimmune diseases including autoimmune/paraneoplastic encephalopathies, bacterial cultures, viral serologies and testing (including HVB, HVC, HIV and COVID-19 in all patients and other serologies driven by presentation and local epidemiology), quantitative immunoglobulins and flow cytometry. Appropriate genetic testing should be considered in patients where there is a suspicious family history of undiagnosed neurological changes or in cases which are resistant to treatment. CSF should undergo evaluation of protein, glucose, immunoglobulins (for evidence of both inflammation and paraneoplastic autoantibodies), oligoclonal bands (in both CSF and serum), IgG index, bacterial/viral testing (including varicella-zoster virus, HSV, syphilis, Lyme disease and tuberculosis testing in all patients, with consideration for other conditions based on local epidemiology), flow cytometry and cytology (performed on serial large-volume samples in the case of low CSF cellularity). Advanced pathogen genetic sequencing techniques, where available, should be considered, if there is persisting uncertainty concerning infection [37].

Patients with evidence of ischaemic stroke on imaging should undergo evaluation for thromboembolic processes, including echocardiography with bubble study, interrogation for arrhythmias, and extracranial vessel imaging for inflammatory and/or atherosclerotic changes. There should be a low threshold for repeat imaging of individuals with acute onset of symptoms or persisting diagnostic uncertainty; resolving stenoses can be diagnostic of reversible cerebral vasoconstriction syndrome, and the lack of contrast enhancement seen in MR-VWI is also suggestive of reversible cerebral vasoconstriction syndrome rather than CNSV [38–40]. In young females being considered for CNSV who present with encephalopathy, vision changes, hearing changes and/or imaging changes of the corpus callosum, ocular fluorescent angiography to assess for Susac syndrome should be considered (clinical vignette 1, see Supplementary Material, available at *Rheumatology Advances in Practice* online) [41]. PET has also been used in some institutions to assess for systemic inflammatory or paraneoplastic processes; however, its utility has yet to be determined. Electroencephalograms can be abnormal but are non-diagnostic; both encephalopathic changes and seizures might be attributable to CNSV or other processes [1, 16].

### Consider a brain biopsy

A brain biopsy is often required to confirm the diagnosis of CNSV: CNSV may not demonstrate radiographic evidence of vasculitis, many mimics may only be differentiated histologically (including intravascular lymphoma, see clinical vignette 2, available at *Rheumatology Advances in Practice* online), and evidence of a neuroinflammatory process may only be evident histopathologically. Clinicians should evaluate patients under the presumption that a brain biopsy is needed to confirm CNSV and take reasonable measures to obtain one; however, there are occasions when it might be infeasible to obtain, such as lesion location or lack of appropriate procedural facilities [2]. Biopsy is ≤75% sensitive for the diagnosis of CNSV; this may be attributable to disease subtype and/or the presence of skip lesions in the parenchyma [42]. Yield can be maximized by ensuring that the biopsy includes cortical, subcortical and leptomeningeal tissue and by targeting areas with either imaging or clinical evidence of disease; areas of parenchyma with abnormalities on MR-VWI are ideal and can increase sensitivity to 89% [8]. When there is no targetable area or there is excess procedural risk, the temporal or non-dominant frontal lobes can be targeted, but they have only 50% sensitivity [43]. This should not dissuade clinicians; a negative biopsy will also lower the probability of an underlying diagnosis of malignancy or other mimics.

Classic biopsy findings in CNSV include parenchymal and/or leptomeningeal vasculitis with transmural mononuclear infiltrates and non-necrotizing granulomas (granulomatous vasculitis), seen in 60% of cases. Twenty percent of cases will each show a lymphocytic infiltrate at least two cells thick (lymphocytic vasculitis) or limited lymphocytic infiltrate with fibrinoid necrosis (necrotizing vasculitis) [44, 45]. A small number of older patients with typically granulomatous vasculitis are also found to have significant deposition of amyloid-β fibrils in the media and adventitia; this is attributable to amyloid-β-related angiitis [46, 47]. These different histological patterns demonstrate the heterogeneity of disease and might represent distinct pathotypes; granulomatous and/or necrotizing vasculitis might connote more severe disease, and amyloid-β-related angiitis might be associated with worse prognosis [48, 49].

## Treatment of CNSV

There are no randomized trials of treatments for CNSV, nor have any consensus guidelines for treatment been published to date; therapy has been inspired by treatment for ANCA-associated vasculitis (AAV) and by treatment for other neuroinflammatory diseases [12, 15, 50]. Given that there have been multiple treatment options with established efficacy for AAV, there is also heterogeneity in how patients with CNSV are treated that are driven by pathological findings, drug availability and severity/phenotype [51]. Initial therapy is guided by disease severity; severe disease is defined by larger volumes of ischaemia and/or the presence of encephalopathy, seizures or organ/function-threatening neurological deficits on presentation. Patients with a large volume of disease, granulomatous/necrotizing angiitis and/or amyloid-β-related angiitis on biopsy are considered to be at the highest risk of poor outcomes [12, 49, 52].

### Immunosuppression in CNSV

Initial therapy for CNSV is high-dose glucocorticoids to rapidly arrest the disease process, which should be started immediately in those with confirmed disease and readily considered where the disease is probable (e.g. abnormal CSF and radiographic findings without a biopsy). For those with severe disease, therapy is typically administered as pulse glucocorticoids (500–1000 mg of i.v. methylprednisolone daily for 3–5 days) followed by high-dose oral glucocorticoids (1 mg/kg, typically 40–60 mg daily). In non-severe cases, oral glucocorticoids can be used without i.v. pulses. The initial dose is administered for 4 weeks before being tapered slowly. There are currently no data demonstrating whether glucocorticoids can be tapered safely more rapidly, as in AAV [53]. Patients should be co-administered a gastric protection agent, sufficient calcium and vitamin D, and there should be an assessment for glucocorticoid-induced osteoporosis.

A second disease-modifying agent, both to provide glucocorticoid-sparing effects and to induce more durable remission, should also be prescribed, stratified by disease severity and patient profile. CYC is the default agent in patients with severe disease; oral formulations continue to be used owing to potential increased efficacy [12, 50, 54, 55]. In those with refractory disease, case reports have demonstrated that rituximab might be effective, although owing to limited access and data, it is not the preferred first-line agent [56, 57]. Patients undergoing induction therapy for severe disease are also typically provided with *Pneumocystis jirovecii* prophylaxis [58]. After an induction phase of ∼6 months, patients are typically switched to an oral immunosuppressant; AZA and mycophenolic acid are preferred. Non-severe disease can be treated with mycophenolic acid, AZA or CYC; MTX is not favoured owing to poor CNS penetrance [30, 59, 60]. IVIG has been used in other vasculitides with conflicting results; given that CNSV predisposes to ischaemic events and there are concerns around potential thromboembolic complications of IVIG, it is generally avoided [61–63].

### Additional therapeutic considerations

Given the neurological deficits associated with CNSV, in addition to its propensity to present with ischaemic stroke, appropriate stroke care is the other important cornerstone of therapy in addition to pharmacotherapy. This includes assessments by speech pathology, physiotherapy, occupational therapy and social work, in addition to provision of supportive devices and modifications to diet, mobility and environment. There is no evidence to support antiplatelet or anticoagulation agents in CNSV; they should be added only if there are other clear indications (e.g. co-morbid atrial fibrillation or secondary prevention of atheroembolic stroke); antiplatelets for secondary prevention of further ischaemic events can also be prescribed. Likewise, seizures should be managed with appropriate anticonvulsants, and neuropsychiatric disturbance should be managed with appropriate psychotropic agents directed by ongoing symptoms.

## Responses to therapy and outcomes

### Response to therapy

Patients should be monitored closely during the induction period for changes, with responses to therapy of CNSV depending on its manifestations of disease. Where there are non-ischaemic neurological deficits, such as headaches and seizures, improvement is typically seen in days to weeks of treatment; patients with strokes typically stop seeing new events within the first 6–8 weeks of therapy. If there are new clinical deficits, the presence of recrudescence and/or ischaemic events attributable to post-inflammatory stenotic changes should be ruled out before additional glucocorticoid therapy is considered. If, however, imaging is typically repeated at ∼8–12 weeks after starting induction therapy to demonstrate response to therapy and again 3–6 months from induction to demonstrate successful remission. Ongoing follow-up depends on disease severity, response and the local availability of imaging; however, imaging should be repeated every 3–6 months with more frequent initial clinical follow-up.

MRA/MRI is the preferred assessment modality because it can capture the vessel lumen, VW and parenchyma. Approximately 50–75% of patients assessed using MR-VWI will show changes in vessel wall enhancement concordant with disease activity; as such, it should again be considered part of a suite of monitoring investigations, if this technique is available at the treatment centre [64]. DSA and CTA might have limited utility because they are invasive, and lumen diameter does not clearly demonstrate treatment response, even with resolution of active inflammation. As such, where MRA/MR-VWI is not available to monitor response to treatment, CTA and DSA will demonstrate a response through the absence of new lesions but cannot assess whether there has been a VW response. Any new areas of stenosis or wall enhancement should be interrogated carefully for possible disease relapse. The optimal method to measure response to treatment is unclear; interpretation of clinical response will be coloured by permanent neurological damage and CSF and radiological investigations have logistic limitations.

### Relapse

It can be difficult to differentiate relapse of CNSV from progression of damage or new insults from atherosclerosis owing to the similarity of presentation. As such, when there is concern for relapse, a comprehensive, multimodal evaluation, including CSF and radiographic investigations, should be performed to demonstrate that changes are attributable to active vasculitis and no other cause, including infection and/or thromboembolic events. Regardless, relapse is common in CNSV and presented in 58 of 191 patients (30%) in one cohort followed over a median of 19 months (range 0–28.1 years) [12]. Treatment of relapse should follow initial treatment, with consideration of pulse or oral glucocorticoids for severe and non-severe relapses and either restarting induction therapy or switching therapeutic agents.

### Outcomes

In addition to the risk of relapse, functional decline owing to damage from CNSV is common, with 70.4% of patients demonstrating some degree of functional impairment attributable to their disease [55]. Although earlier diagnosis and aggressive therapy have improved outcomes, mortality from CNSV seen in 11–28% over long-term follow-up, typically seen in the first year [12, 55]. Although not well reported in CNSV, it is likely that there is also significant morbidity associated with treatment, including cardiovascular risk, infections and osteoporosis [65]. Risks of disease and therapy must be balanced, and as such, treatment durations for CNSV are unclear. Currently, there are no data to suggest whether there is an appropriate duration of immunosuppression in CNSV, and there is significant variation in practice in terms of the duration of therapy, ranging from 5 years to lifelong immunosuppression based on the individual patient profile. It is possible that patients with non-severe, monophasic disease might be treated safely with shorter durations of therapy; those who relapse typically have prolonged treatment duration.

## Future directions

This review synthesizes expert knowledge concerning CNSV and also demonstrates that there are significant gaps across diagnosis, treatment, monitoring and outcomes. The foremost of these to address are as follows: establishing an effective diagnostic pathway of CNSV; understanding the strengths and limitations of the proposed diagnostic criteria; understanding whether subtypes represent disease endotypes (potentially based on clinical/vessel phenotype, disease severity and/or histologic findings); establishing consensus recommendations for treatment of CNSV; determining optimal methods of assessing responses to therapy; and understanding predictors of relapse and outcomes to assist in clinical decision-making.

The rarity and poor awareness of CNSV have historically been the greatest barriers in carrying out clinical research in the disease space. The evolution and proliferation of imaging, however, have significantly assisted in disease recognition, diagnosis and monitoring of CNSV, and increased interest has led to wide reporting concerning the disease, although single-centre cohorts will be subject to selection bias, clinician preferences and the availability of local investigations and treatment [16]. Other rare diseases, most analogously AAV, have demonstrated that through collective interest and collaboration, large prospective international cohorts of individuals aggregated and diseases previously considered rare are now more easily studied. Indeed, during the last 20 years, international research consortiums, including the Vasculitis Clinical Research Consortium (VCRC) and the European Vasculitis Society, have realized marked advances in caring for patients with vasculitides, including AAV and GCA.

Similar steps are now being taken for primary CNSV. An international prospective cohort of patients with CNSV is underway to collect a wide variety of data, including: clinical and radiological findings; longitudinal disease and therapeutic outcomes; and collection of biological specimens for analysis of possible new biomarkers of disease. Through this cohort, disease definitions can be refined, and a comprehensive theory of disease can be constructed; early priorities are to develop consensus approaches to diagnosis, to establish unbiased phenotypes and presentations of disease, and evolve collaborations to develop a platform for further research. This platform can then be used to explore and validate new biomarkers of disease, design clinical trials of new therapies or better establish the efficacy of existing agents and improve our understanding of disease outcomes to inform clinical decision-making.

## Supplementary Material

rkad080_Supplementary_Data

## Data Availability

No new data were generated or analysed in support of this research.
