# Supplementary material for: Current and future advances in practice: a practical approach to the diagnosis and management of primary central nervous system vasculitis
Source: Rheumatol Adv Pract. 2023 Nov 20;7(3):rkad080. doi: 10.1093/rap/rkad080 (PMC10712448; doi:10.1093/rap/rkad080)
Supplement: rkad080_Supplementary_Data [file rkad080_supplementary_data.docx]

**Supplementary material**

**Clinical Vignette 1: Susac Syndrome**

A 32-year-old woman with no past medical history presented with multiple weeks of headaches dysarthria, confusion, bilateral arm weakness and right arm numbness. She underwent thorough work up including multiple imaging and laboratory studies with no evidence of systemic inflammatory, infectious or embolic diseases. Cerebrospinal fluid revealed elevated white blood count of 27 cells/microliter and elevated protein of 101mg/dl. Sagittal FLAIR images on MRI revealed (A) linear ‘icicle’ lesions in corpus callosum with multiple micro infarcts on diffuse weighted images with (B) scattered foliar leptomeningeal enhancement on T1 post SPACE imaging (C). Cerebral digital subtraction angiography was normal. Giving the finding of corpus callosum lesions, fluorescein angiography was performed and revealed branch retinal artery occlusion (d). The diagnosis of Susac syndrome was confirmed and patient was started on treatment.

| 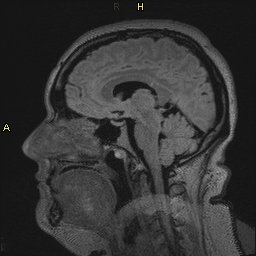 | 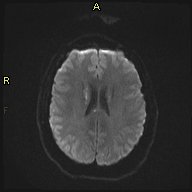 |
| --- | --- |
| A) “Icicle” lesions on the corpus callosum on FLAIR MRI sequencing | B) Foliar leptomeningeal enhancement on T1 MRI sequencing |
| 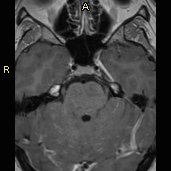 | 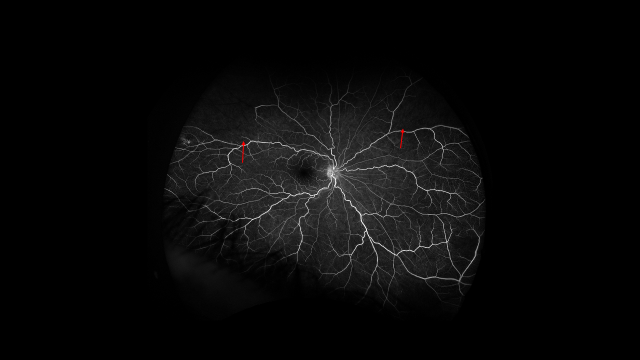 |
| C) Foliar leptomeningeal enhancement on SPACE MRI sequencing | D) Peripheral non perfusion due to branch retinal artery occlusion on fluorescein angiography |

**Clinical Vignette 2: Intracranial lymphoma**

A 66-year-old male with Crohn’s disease, maintained on vedolizumab and variable dose of prednisone with minimum of 10 mg daily, presented with a 9-month history of new cerebral infarcts across multiple vascular territories despite anticoagulation and cardiovascular risk optimization. CSF revealed elevated protein (147 mg/dl, normal <45) but no nucleated cells nor evidence of infection. Assessment for thromboembolic causes demonstrated a non-contributory patent foramen ovale but was otherwise unrevealing. Axial FLAIR MRI revealed scattered hyperintensities (A) of different ages along with multiple infarcts of varying ages with diffusion-weighted MRI (B). Cerebral angiography revealed stenoses across all vascular territories including the anterior communicating artery aneurysm (C). CNS vasculitis was suspected, however these stroked occurred despite immunosuppression with variable doses of glucocorticoids for his Crohn’s disease, which prompted further evaluation with brain biopsy. Pathology revealed venous-type blood vessel luminally congested with atypical cells, consistent with intravascular lymphoma with positive CD20 stain for B cell lymphocyte marker (D). The patient was ultimately diagnosed with intravascular intracranial lymphoma.

| 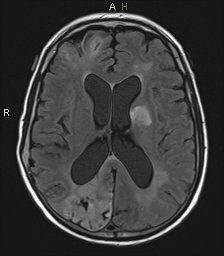 | 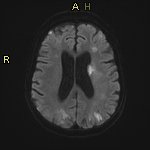 |
| --- | --- |
| A) FLAIR hyperintensities of varying age | B) DWI changes of varying age |
| 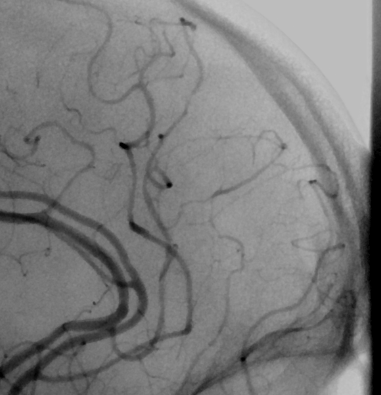 | 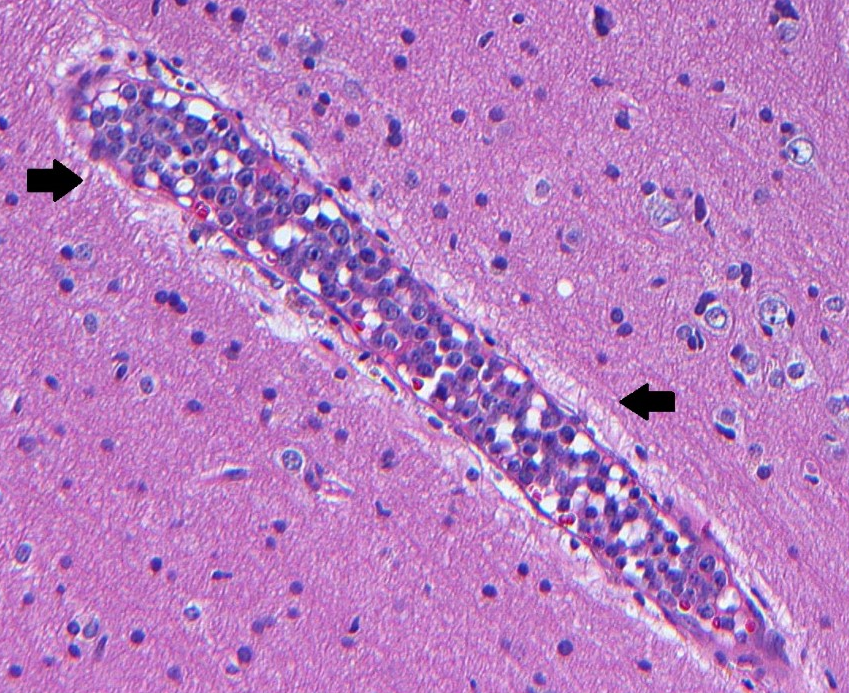 |
| C) Angiography with multiple luminal occlusions | D) Histology demonstrating vessels congested with CD20 lymphocytes |
